# Neurodegenerative Disease and the NLRP3 Inflammasome

**DOI:** 10.3389/fphar.2021.643254

**Published:** 2021-03-10

**Authors:** Jonathan A. Holbrook, Heledd H. Jarosz-Griffiths, Emily Caseley, Samuel Lara-Reyna, James A. Poulter, Caroline H. Williams-Gray, Daniel Peckham, Michael F. McDermott

**Affiliations:** ^1^ Department of Clinical Neurosciences, John Van Geest Centre for Brain Repair, University of Cambridge, Cambridge, United Kingdom; ^2^ Leeds Institute of Rheumatic and Musculoskeletal Medicine (LIRMM), University of Leeds, Leeds, United Kingdom; ^3^ Leeds Institute of Medical Research at St. James’s University Hospital, Leeds, United Kingdom; ^4^ Leeds Cystic Fibrosis Trust Strategic Research Centre, University of Leeds, Leeds, United Kingdom; ^5^ Institute of Microbiology and Infection, University of Birmingham, Birmingham, United Kingdom; ^6^ Leeds Centre for Cystic Fibrosis, St James’s University Hospital, Leeds, United Kingdom

**Keywords:** neurodegenerative disease, NLRP3 inflammasome, Alzheimer's disease, Parkinson’s disease, neuroinflammation, inflammation

## Abstract

The prevalence of neurodegenerative disease has increased significantly in recent years, and with a rapidly aging global population, this trend is expected to continue. These diseases are characterised by a progressive neuronal loss in the brain or peripheral nervous system, and generally involve protein aggregation, as well as metabolic abnormalities and immune dysregulation. Although the vast majority of neurodegeneration is idiopathic, there are many known genetic and environmental triggers. In the past decade, research exploring low-grade systemic inflammation and its impact on the development and progression of neurodegenerative disease has increased. A particular research focus has been whether systemic inflammation arises only as a secondary effect of disease or is also a cause of pathology. The inflammasomes, and more specifically the NLRP3 inflammasome, a crucial component of the innate immune system, is usually activated in response to infection or tissue damage. Dysregulation of the NLRP3 inflammasome has been implicated in the progression of several neurodegenerative disorders, such as Alzheimer’s disease, Parkinson’s disease, Huntington’s disease, amyotrophic lateral sclerosis, and prion diseases. This review aims to summarise current literature on the role of the NLRP3 inflammasome in the pathogenesis of neurodegenerative diseases, and recent work investigating NLRP3 inflammasome inhibition as a potential future therapy.

## Introduction

**FIGURE 1:**
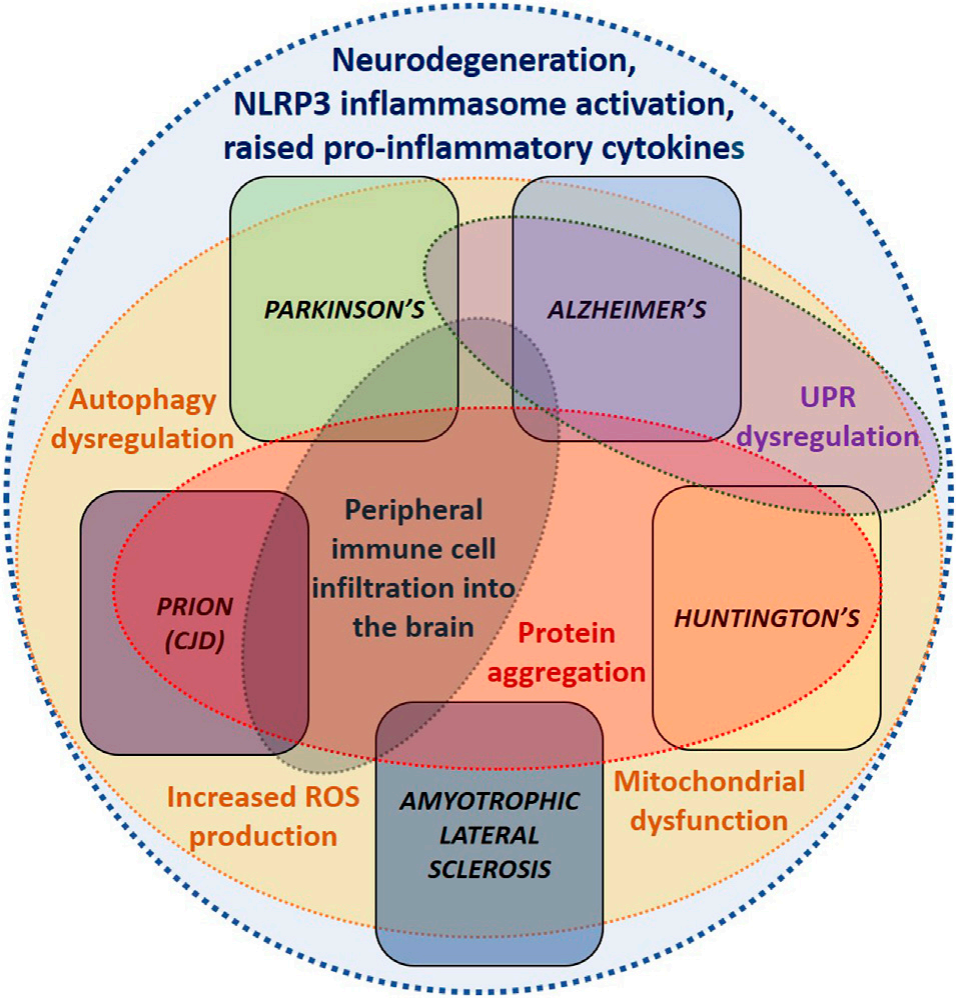
Common dysregulated mechanisms underlying neurodegenerative diseases. Demonstrating the similarities between the dysregulated mechanisms underlying each of the neurodegenerative diseases covered in this review. These overlapping disease mechanisms suggest that similar therapeutics could be utilised to treat several of these conditions.

Neurodegenerative disease is an increasingly common societal issue, especially in countries with an aging population. These diseases are heterogeneous in their clinical presentations (Table 1), with a diverse range of underlying mechanisms, resulting in a variety of underlying pathophysiologies (Dugger and Dickson, 2017). However, despite their heterogeneous nature, systemic activation of the immune system remains a common feature that is implicated in the progression of many of these diseases (Figure 1) (Amor et al., 2010). This is further complicated by the observation that immune activation can function as a double-edged sword, whereby in some contexts it acts as an aid to cellular repair and regeneration, such as clearance of debris by microglia (Jin and Yamashita, 2016), whereas in others, it may be detrimental (Kempuraj et al., 2016). Furthermore, the central nervous system (CNS) has, until relatively recently, been considered an immune privileged site; however, the discovery of a functional meningeal lymphatic system, that allows movement of cerebrospinal fluid (CSF) to the cervical lymph nodes, has challenged this view and further emphasised the importance of the immune system in the pathology of neurodegenerative disease (Louveau et al., 2015).

**TABLE 1:** Neurodegenerative disease. Showing typical age of onset, symptoms and primary areas of central nervous system (CNS) involvement.

Disease	Typical age of onset	Global prevalence	Symptoms	Primary area of CNS effected
Alzheimer’s	∼65, but early onset <50	∼712 per 100,000	Episodic memory deficits, apathy and depression are early symptoms. Later symptoms include impaired communication, disorientation, confusion, poor judgment, behavior changes and, ultimately, difficulty speaking, swallowing and walking	Entorhinal cortex, hippocampus, cerebral cortex
Parkinson’s	∼60, but early onset <50	∼160 per 100,000	Bradykinesia, muscle rigidity, tremors, impaired posture and balance. Non-motor disturbances such as motivation and memory	Substantia nigra
Huntington’s	∼35	∼2.7 per 100,000	Progressive chorea, cognitive decline and psychiatric disorders.	Basal ganglia and cerebral cortex
Amyotrophic lateral sclerosis	∼65	∼2.2 per 100,000	Muscle weakness and progressive paralysis, respiratory insufficiency	Brain stem, spinal cord and primary motor cortex
Prion diseases	∼60	∼0.1–0.2 per 100,000	Loss of intellect and memory, personality changes, slurred speech, loss of balance/coordination, vision problems, abnormal jerking movements, progressive cognitive impairment and mobility	Cerebral cortex

**FIGURE 2:**
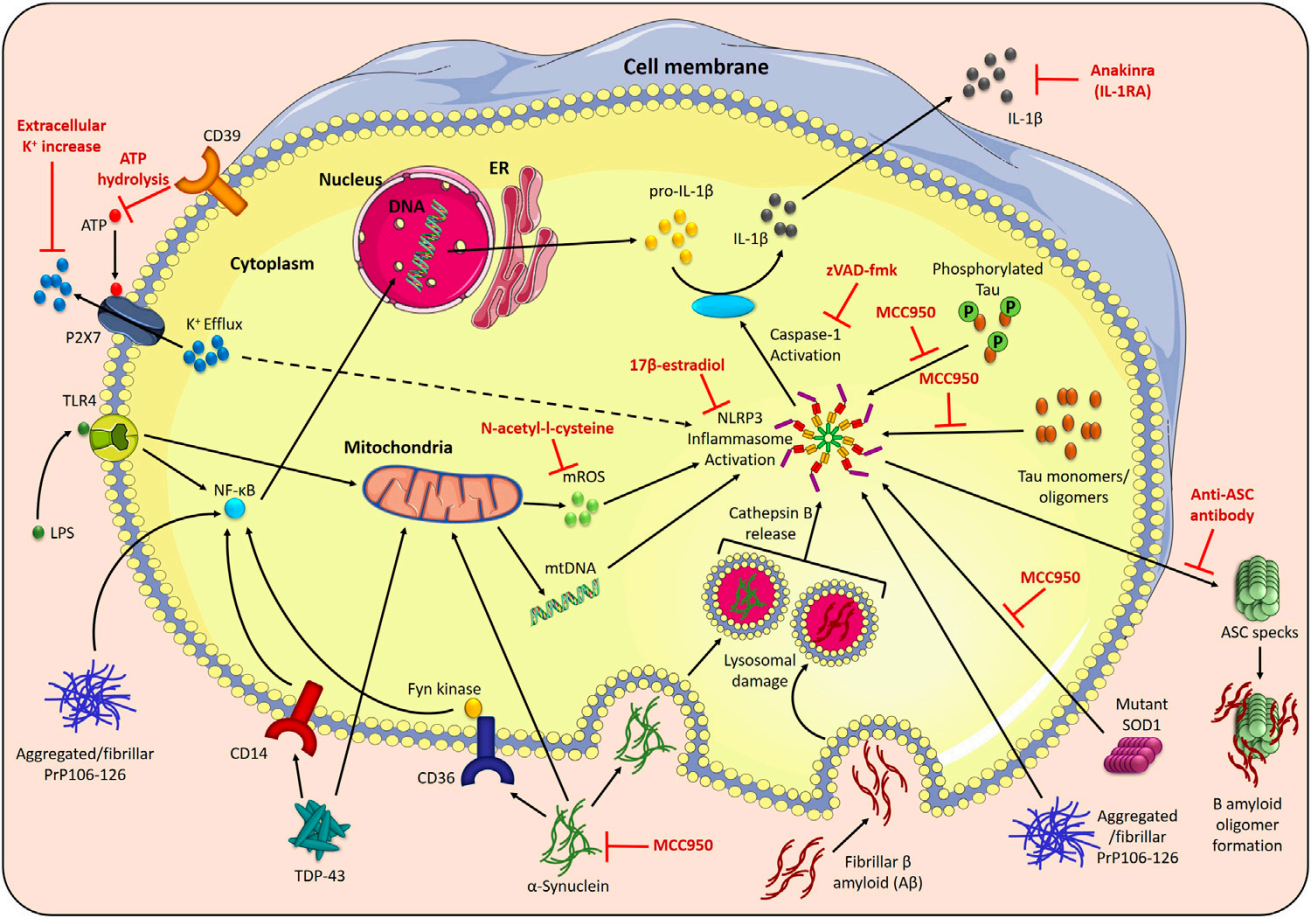
NLRP3 inflammasome activation in neurodegenerative disorders. The NLRP3 inflammasome assembles in response to two signals; toll-like receptor 4 (TLR4) stimulation by LPS induces the NF-κβ-mediated transcription of pro-IL-1β and pro-IL-18, and stimuli such as P2X7 receptor-facilitated potassium (K^+^) efflux trigger NLRP3 inflammasome activation. The activated NLRP3 inflammasome recruits an adaptor protein, apoptosis-associated speck-like protein containing a CARD (ASC), and oligomerises to activate caspase-1, which cleaves pro-IL-1β and pro-IL-18 into their active forms. Disease-related proteins can also activate the NLRP3 inflammasome. Phagocytosis of fibrillar β-amyloid (Aβ) (Alzheimer’s disease) causes NLRP3 inflammasome activation via lysosomal damage and cathepsin B release. Aβ also binds to ASC specks released during inflammasome activation, increasing the formation of Aβ oligomers. Tau monomers and oligomers (Alzheimer’s disease) activate the NLRP3 inflammasome, which, in turn, affects tau hyperphosphorylation and aggregation. Phagocytosis of aggregated α-synuclein (Parkinson’s disease) results in NLRP3 inflammasome activation, and uptake of aggregated α-synuclein, mediated by Fyn kinase and the class B scavenger receptor CD36, facilitates LPS-independent inflammasome priming. α-synuclein is also associated with mitochondrial dysfunction, including mitochondrial DNA (mtDNA) disturbances. Mutant SOD1 (ALS) acts as a DAMP to activate the NLRP3 inflammasome. Transactive response DNA-binding protein-43 (TDP-43) (ALS) causes CD14-induced NF-κβ activation, and triggers mtDNA release. In prion diseases, aggregated and fibrillar forms of the neurotoxic PrP-derived peptide (PrP106–126) are involved in NLRP3 inflammasome priming, via triggering NF-κβ signaling, and activation. This NLRP3 inflammasome activation can be prevented by the NLRP3 specific inhibitor, MCC950, increasing the extracellular K^+^ concentration to prevent K^+^ efflux, prevention of P2X7 receptor activation by CD39-mediated ATP hydrolysis, inhibition of mitochondrial reactive oxygen species (mROS) by N-acetyl-l-cysteine and the anti-inflammatory hormone 17β-estradiol. The broad caspase inhibitor, zVAD-fmk, and the recombinant IL-1 receptor antagonist, anakinra, both reduce the downstream effects of NLRP3 activation in disease.

The NLRP3 inflammasome is a multimeric protein complex, which assembles in response to homeostasis-altering molecular patterns (HAMPs), pathogen-associated molecular patterns (PAMPs) and danger-associated molecular patterns (DAMPs), and functions as a centrally important component of the innate immune system (Figure 2) (Martinon et al., 2002; Liston and Masters, 2017; Kelley et al., 2019). It consists of three main components: an apoptosis-associated speck-like protein containing a CARD (caspase activation and recruitment domain) (ASC), which functions as a central adaptor protein; an inflammatory caspase, caspase-1, and a pattern recognition receptor (PRR) protein, NLRP3 (nucleotide-binding domain (NOD)-like receptor protein 3) (Kelley et al., 2019). There are several different inflammasomes, all defined by the PRRs they contain; however, this review will focus specifically on the NLRP3 inflammasome (Zheng et al., 2020). Upon activation, via detection of PAMPs or DAMPs, these various components undergo conformational change to subsequently assemble and nucleate the oligomerisation of monomeric PRR proteins (Lu et al., 2014) and form the NLRP3 inflammasome. This large multimeric protein comple acts via caspase-1 dependent proteolytic cleavage of several proteins, including pro-interleukin (pro-IL)-18 and pro-IL-1β to their mature inflammatory cytokines, IL-18 and IL-1β (Kelley et al., 2019). IL-18 is important for interferon-γ (IFNγ) production as well as negative regulation of the Th17 cell population and promotion of key Treg cell generation, thereby playing an important role in the regulation of intestinal inflammation and adaptive immunity (Harrison et al., 2015). By contrast, IL-1β induces fever, sensitises neutrophils to chemoattractants, stimulates vasodilation, and increases the expression of adhesion molecules, thereby facilitating the infiltration of immune cells into damaged or infected tissues (Kelley et al., 2019). Furthermore, gasdermin D (GSDMD) also undergoes NLRP3 inflammasome-dependent cleavage, which facilitates GSDMD’s insertion into cellular membranes to form pores, thus initiating a specific kind of cell death called pyroptosis (Fink and Cookson, 2006).

In recent years, our understanding of NLRP3 inflammasome activation and regulation has rapidly progressed, leading the way to new developments in potential therapeutics for several autoinflammatory diseases (Caseley et al., 2020). This review aims to summarise recent progress in our understanding of several neurodegenerative diseases, whose development and/or progression has been linked to inflammation involving activation of the NLRP3 inflammasome. The review also considers the use of novel therapeutics to target NLRP3 inflammasome activation, with the possibility of modifying the clinical course of these disorders (Table 2).

**TABLE 2:** NLRP3 inhibitors and possible mechanisms of action.

NLRP3 inflammasome inhibitor compounds	Possible mechanism of action	Neurodegeneration research
MCC950	Walker B motif interaction and inhibition of ATP hydrolysis	Fiala et al. (2002), Heneka et al. (2013), Liu and Wang (2017), Flores et al. (2018), Tzeng et al. (2018), Antonyová et al. (2020)
Anti-ASC antibody	Directly binds to ASC specks	Daniels et al. (2016)
Anakinra	Directly binds to IL-1 receptor, blocking IL-1β function	Kadhim et al. (2016), Meissner et al. (2010)
17β-estradiol	Unknown	Bellezza et al. (2018)
Dopamine	Binds to dopamine D1 receptor, inducing ubiquitination of NLRP3	Rushworth et al. (2013)
Kaempferol	NF-κB inhibition and ASC oligomerisation inhibition	Elliott et al. (2018)
VX-765	Inhibits caspase-1	Venegas et al. (2017)
zVAD-fmk	Inhibits caspase-1	Zhao et al. (2017)

## Alzheimer’s Disease

Alzheimer’s disease (AD) is the most common progressive age-associated neurodegenerative disorder, characterised by abnormal accumulation of protein aggregates in the form of β-amyloid (Aβ)-containing plaques, and neurofibrillary tangles composed of hyperphosphorylated tau (Blennow et al., 2006; De Strooper and Karran, 2016). These protein aggregates are at first found in the neocortex, but, over time, progress to the entorhinal cortex and hippocampus (Thal et al., 2002) (Table 1). The accumulation of aggregated protein, as in many other neurodegenerative diseases, coincides with the activation of microglia and astrocytes, which promote the release of inflammatory molecules to aid cellular repair, as well as phagocytosis of unwanted debris. However, sustained activation of microglia and higher expression of inflammatory molecules, particularly IL-1β, has been found in microglial cells surrounding Aβ plaques, in AD patients as well as animal models of disease (Figure 2) (Griffin et al., 1989; Simard et al., 2006). Moreover, increased levels of IL-1β and IL-18 have also been detected in the CSF of patients with AD (Blum-Degen et al., 1995). There is evidence to suggest that both aggregated protein accumulation and immune cell over-activation can compromise the structure and function of neurons, resulting in episodic memory deficits and cognitive impairment, characteristic of AD (Blennow et al., 2006; Ising and Heneka, 2018; Feng et al., 2020).

The NLRP3 inflammasome has been implicated as a key commonent of the innate immune response in AD. The evidence discussed below provides a clear basis for NLRP3 inflammasome inhibition/modulation to be considered as a therapeutic strategy to delay progression of this disease. Cultured monocytes, isolated from patients with AD, were reported to have increased gene expression of *NLRP3* as well as *ASC*, *caspase-1,* and the cytokines, *IL-1β* and *IL-18* (Saresella et al., 2016), which suggests that the peripheral NLRP3-mediated immune response is increased in disease. In line with this, there is evidence to suggest that peripheral monocytes can infiltrate the CNS in AD and accumulate near areas of pathology (Figure 1) (Fiala et al., 2002). Using *in vitro* models, activation of the NLRP3 inflammasome was found to be initiated after fibrillar Aβ was phagocytosed by microglia, leading to lysosomal damage with cathepsin B release, caspase-1 activation and release of IL-1β as well as TNF and nitric oxide (Halle et al., 2008). Consistent with these observations, Henske et al., 2013 reported an increased amount of active caspase-1 in brain lysates from AD patients with mild cognitive impairment (MCI), relative to healthy controls, suggestive of chronic inflammasome activation. In the same study, using a transgenic mouse model of AD, aged amyloid precursor protein (APP)/presenilin (PS1)M146V mice were also found to have increased active caspase-1 levels. The genetic ablation of NLRP3 or caspase-1 protected the APP/PS1 mice from spatial memory deficits, reducing brain levels of caspase-1 and IL-1β, as well as enhancing microglial phagocytic ability, thereby increasing Aβ clearance (Heneka et al., 2013). Interestingly, NLRP3 inflammasome deficiency also skewed microglial cells toward an M2 (anti-inflammatory) phenotype, which correlated with decreased deposition of Aβ, suggesting that microglia with an M2 phenotype may play a protective role in AD (Heneka et al., 2013). Furthermore, long-term neuronal changes in a mouse AD model, after acute peripheral immune stimulation, were shown to be both NLRP3 inflammasome and age-dependent (Beyer et al., 2020). In another study, ASC specks, which are pivotal components of the NLRP3 inflammasome, are released by microglia during pyroptosis, and rapidly bind to Aβ, increasing Aβ-oligomer formation and the spread of Aβ pathology in APP/PS1 mice. ASC-deficient APP/PS1 mice, as well as those administered with anti-ASC antibody, were able to block the increase in Aβ pathology (Figure 2) (Venegas et al., 2017). Collectively, these findings indicate that amyloid can directly activate microglial NLRP3 inflammasome, which triggers the chronic release of pro-inflammatory cytokines and ASC specks, and promote the development of AD pathology.

Direct inhibition of the NLRP3 inflammasome with the small molecule inhibitor, MCC950, also known as CRID3, improved cognitive function and reduced Aβ accumulation, as well as promoting Aβ clearance in APP/PS1 mice (Figure 2) (Dempsey et al., 2017). Triple-transgenic (3 × Tg)AD mice provide a unique model of AD, as they contain the human APP Swedish mutant transgene, tau (P301L), and PS1(M146V) knock-in mutation. Indirect inhibition of NLRP3 inflammasome activation in 3 × TgAD mice using the fenamate non-steroidal anti-inflammatory drug, mefanamic acid, completely abrogated the AD-related neuroinflammation, with levels of IL-1β expression and microglial activation reduced to wild-type levels. Fenamates suppress chloride efflux via the volume regulated anion channel (VRAC), which, in turn, blocks NLRP3 activation and IL-1β release (Daniels et al., 2016). In another study, inhibition of caspase-1 activity with VX-765 dose-dependently reversed episodic and spatial memory impairment and reversed brain inflammation and Aβ deposition in the J20 mouse model of AD (Flores et al., 2018). Interestingly, although NLRP3 deletion and caspase-1 inhibition appears to protect against amyloid-induced AD-like disease, IL-18 deletion did not protect APP/PS1 mice. Instead, IL-18-deficient AD mice were more susceptible to aberrant neuronal transmission in AD (Tzeng et al., 2018).

The impact of tau on activation of the NLRP3 inflammasome hasn’t been explored to the same extent as Aβ, but a recent article by Stancu et al., in 2019 demonstrated that tau seeds are able to activate the NLRP3 inflammasome in primary microglia, and that ASC deficiency in tau transgenic mice inhibited the seeding of tau pathology. Intracerebral administration of MCC950 inhibited exogenously seeded tau pathology (Heneka et al., 2013; Stancu et al., 2019). In another study, tau monomers and oligomers were found to activate the NLRP3 inflammasome, an effect which could be inhibited by MCC950 (Ising et al., 2019). The loss of NLRP3 inflammasome function reduced tau hyperphosphorylation and aggregation, a phenomenon which could be attributed to IL-18, a known inducer of kinases, Cdk5 and glycogen synthase kinase-3β (GSK-3β), which are involved in the hyperphosphorylation of tau (Ojala et al., 2008). Tau monomers/oligomers could therefore activate the NLRP3 inflammasome, and subsequent injection with fibrillar Aβ-containing brain homogenates could induce tau seeding and pathology. Together, these data suggest that neurofibrillary tangles develop downstream of Aβ-induced microglial activation (Ising and Heneka, 2018).

Previous studies have described the pathogenic role of IL-1β in AD, with IL-1β cerebral injection raising amyloid precursor protein (APP) levels in wild-type mice (Sheng et al., 1996). In mice with a deficiency of IL-1Ra, subsequent intra-cerebroventricular injection with oligomeric Aβ1-42 resulted in mice which were more vulnerable to Aβ oligomers (Craft et al., 2005). However valid, these studies were based on acute injection of IL-1β or Aβ oligomers and only provide indirect evidence of the involvement of IL-1 signaling in AD. It is likely that in AD, the increase in IL-1β is a chronic process which develops over months/years (Shaftel et al., 2007). Shaftel et al., in 2007, found that sustained over-expression of IL-1β in APP/PS1 mice reduced plaque pathology, possibly due to increased phagocytic activity of microglia and macrophages (Shaftel et al., 2007). In a later study, although sustained expression of IL-1β reduced amyloid load in 3xTgAD mice, kinases and phosphatases, involved in tau phosphorylation, were found to be increased (Ghosh et al., 2013). Therefore, while IL-1β may be beneficial in the first instance to clear amyloid deposition, the underlying increase in tau phosphorylation may drive both tau and amyloid pathology, via activation of the NLRP3 inflammasome. These data are consistent with other investigations whereby modulation of innate immunity *in vivo* caused a reduction of Aβ, with a corresponding increase in microglial activation. For example, intracranial administration of lipopolysaccharide (LPS) in a mouse model of AD leads to a reduction in amyloid pathology through microglial clearance of Aβ (DiCarlo et al., 2001; Herber et al., 2004). A recent study by Wendeln et al., in 2018, explored whether peripheral stimulation of LPS could trigger innate immune memory in brain microglia. Using the APP23 murine AD model, which develops insoluble amyloid plaques at 6 months of age, subcutaneous injection of a low dose of LPS at 3 months of age increased the number of amyloid plaques at 6 months, whereas a 4 x dose of LPS decreased the number of plaques, confirming that peripheral immune stimuli can cause long-term alterations in brain innate immune response, and can differentially affect the development of Alzheimer’s pathology (Wendeln et al., 2018). Trained immunity in the brain is a very interesting phenomenon and, given that both infections and diseases, such as diabetes or arthritis, are associated with chronic inflammatory processes and are considered as risk factors for Alzheimer’s disease, epigenetically-modified microglia could provide one possible explanation for this effect.

## Parkinson’s Disease

Parkinson’s disease (PD) is the second most common neurodegenerative disease, with about 6 million people affected worldwide (GBD, 2019). The characteristic pathophysiology of PD involves loss of dopaminergic neurons in the substantia nigra (SN) pars compacta resulting in a lack of dopamine in the nigrostriatal system. This results in motor symptoms including bradykinesia, muscle rigidity, and tremors, as well as other non-motor disturbances such as loss of motivation and low mood, which tend to respond well to dopamine-replacing therapies (Massano and Bhatia, 2012) (Table 1). However, CNS pathology in PD also extends outside of the dopaminergic nigrostriatal system and involves widespread non-dopaminergic pathways (Pfeiffer, 2016). This leads to a number of other clinical features, such as dementia and balance problems, which are unresponsive to dopamine replacing therapies (Chaudhuri and Schapira, 2009). Neuronal dysfunction in PD, in both subcortical and cortical regions, is accompanied by intracellular aggregation of the α-synuclein protein to form Lewy bodies. The precise role of these protein aggregates in cell dysfunction, and why they form is still not fully known, but a complex interplay of both environmental and genetic factors are implicated, which impact on several essential cellular processes, including mitochondrial physiology, lysosomal function and autophagy (Figure 1) (Kitada et al., 1998; Valente et al., 2004; Angeles et al., 2011; Wang et al., 2012; Kalia and Lang, 2015; Chang et al., 2017; Alessi and Sammler, 2018).

Immune dysregulation has also been strongly implicated in PD pathogenesis (Gao and Hong, 2008; Williams-Gray et al., 2018), both systemically and within the central nervous system (Tan et al., 2020). Genome-wide association studies (GWAS) have reported a number of genetic variants, which are associated with an increased risk of developing PD (Zabetian et al., 2007; Pankratz et al., 2009; Satake et al., 2009; Simon-Sanchez et al., 2009; Edwards et al., 2010; Hamza et al., 2010; Saiki et al., 2010; Nalls et al., 2014; Pierce and Coetzee, 2017).

In PD, activated microglia have been found in the SN, and also in more widespread subcortical and cortical regions (McGeer et al., 1988; Imamura et al., 2003; Kouli et al., 2020), and are implicated in neuronal toxicity, via secretions of inflammatory cytokines as well as inducing astrocytes to release neurotoxic elements (Liddelow et al., 2017). Furthermore, in patients with PD, a pro-inflammatory profile of immune markers in the serum at diagnosis is linked to a faster subsequent decline in motor function and lower cognitive scores (Williams‐Gray et al., 2016). α-synuclein may play a critical role in driving peripheral immune activation in PD (Sulzer et al., 2017; Scott et al., 2018; Schonhoff et al., 2020; Wijeyekoon et al., 2020), which may be associated with faster disease progression, presumably due to peripheral immune cells and cytokines crossing the blood brain barrier to promote microglial activation and neurotoxicity (Figure 1) (Kouli et al., 2020).

Similar to AD research, in recent years, there is mounting evidence to specifically implicate the NLRP3 inflammasome in PD disease progression. In human post-mortem brain from PD cases, NLRP3 expression is elevated in mesencephalic neurons; furthermore, *NLRP3* genetic polymorphisms are associated with downregulation of NLRP3 activity and reduced risk of PD (von Herrmann et al., 2018). *In vitro* work suggests possible mechanistic links between NLRP3 activation and α-synuclein aggregation; specifically, activation of the NLRP3 inflammasome in the neuronal cell line, BE(2)-M17, that overexpresses α-synuclein, leads to aggregation of α-synuclein, which is preventable by inhibition of caspase-1 (Figure 2) (Wang et al., 2016). It was also found that, caspase-1 cleaves α-synuclein at Asp121 *in vitro,* thereby predisposing α-synuclein to aggregate. Moreover, caspase-1 has been reported to co-localise with α-synuclein in post-mortem PD brains (Wang et al., 2016). Interestingly, caspase-1 and α-synuclein levels are also highly correlated in human serum, and both are lower in PD than controls, suggesting that they may be co-sequestered out of serum into intracellular aggregates (Wijeyekoon et al., 2020). It has also been reported that newly diagnosed PD patients have increased systemic IL-1β levels in the serum (Williams‐Gray et al., 2016), and systemic NLRP3 inflammasome expression and activation are correlated with motor severity and progression in PD (Fan et al., 2020). Furthermore, human monocytes have also been shown to phagocytose aggregated α-synuclein, which leads to a more pronounced NLRP3 inflammasome response (Codolo et al., 2013). Fyn kinase, in conjunction with CD36, regulates microglial uptake of aggregated α-synuclein thereby linking Fyn kinase and CD36 activity to NLRP3-driven inflammation (Panicker et al., 2019). Interestingly, Fyn kinase is also involved in the phosphorylation of Tau at Tyr18 and is present in neurofibrillary tangles in AD (Lee et al., 2004). Multiple reports show Aβ-induced synaptic dysfunction involving the tau-Fyn axis (Larson et al., 2012; Um et al., 2012; Um and Strittmatter, 2013; Frandemiche et al., 2014; Nygaard, 2018); specifically binding of Aβ oligomers to the cellular prion protein (PrP^C^) on the surface of neurons directly activates Fyn kinase, which in turn phosphorylates tau (Rushworth et al., 2013). Fyn has also been implicated as a key regulator of tau pathology independently of Aβ-induced toxicity (Briner et al., 2020). A recent report found that OLT1177, a β-sulfonyl nitrile molecule, is a selective inhibitor of the NLRP3 inflammasome, and in fact reduces Fyn kinase levels by 35% in human monocyted derived macrophages following stimulation with LPS and nigerecin (Marchetti et al., 2018), and thus could be beneficial in both AD and PD.

Mitochondrial dysfunction, such as reduced activity of mitochondrial electron transport chain complex I, mutations in mitochondrial quality control genes, and mtDNA disturbances has been implicated in the pathogenesis of PD (Bose and Beal, 2016; Matheoud et al., 2016; Antonyová et al., 2020). Mitochondria are also key regulators of the NLRP3 inflammasome, with mitochondrial dysfunction resulting in NLRP3 assembly and activation (Zhou et al., 2011; Elliott et al., 2018). Impairment of mitochondrial function in microglia has been found to amplify NLRP3 inflammasome activity (Sarkar et al., 2017), with the NLRP3 inflammasome being highly expressed in activated microglia, in post mortem PD brains. Neurotoxins, aggregation of α-synuclein, mitochondrial reactive oxygen species (mROS), and dysregulated mitophagy are all key regulators of NLRP3 inflammasome activation, leading to IL-1β and IL-18 release as well as pyroptotic cell death of neurons in the SN (Wang et al., 2019; Haque et al., 2020). Furthermore, a recent study demonstrated that the stimulation of mitophagy, in a murine 1-methyl-4-phenyl-1, 2, 3, 6-tetrahydropyridine (MPTP) induced PD model, in an attempt to aid in clearance of damaged mitochondria suppressed NLRP3 inflammasome activation in microglia, reducing inflammation, dopaminergic neuronal loss and improving behavioral parameters (Ahmed et al., 2020).

It is worth noting that dopamine has been reported to inhibit NLRP3 inflammasome activation via the dopamine D1 receptor (DRD1), as DRD1 signaling induces the binding of ubiquitin to NLRP3, promoting its degradation (Yan et al., 2015). Hence loss of dopamine in PD may facilitate NLRP3 activation, but, conversely, dopamine-replacing medication may act to suppress this. Other regulators of NLRP3 inflammasome activation, with implications for PD etiology, include the long noncoding RNA, lncRNA-Cox2, which regulates both autophagy and microglial NLRP3 inflammasome activation via binding to NF-κB, inducing its translocation to the nucleus and upregulation of NLRP3 related genes (Xue et al., 2019). The knockdown of lncRNA-Cox2 in microglia has been reported to reduce NLRP3 inflammasome activation and IL-1β secretion (Xue et al., 2019). Also, the microbiota-gut-brain axis has been implicated in the development of PD via enteric bacterial regulation of the NLRP3 inflammasome (Pellegrini et al., 2020), with heightened IL-1β mRNA expression in the colon (Devos et al., 2013). Gut inflammation may be an important driver of the systemic immune response in PD.

This accumulating evidence suggests that specific modulation of the NLRP3 inflammasome may be a promising therapeutic target in PD (Haque et al., 2020). Indeed, NLRP3 inhibitors, such as MCC950, simultaneously reduce microglial activation, motor deficits, SN dopaminergic degeneration and accumulation of α-synuclein aggregates, upon oral administration in mice that have undergone injection of fibrillar α-synuclein in the striatum (Gordon et al., 2018). Furthermore, NLRP3 inflammasome-active microglia lead to neuronal cell death in a murine MPTP-induced PD model (Lee et al., 2019), with KO of NLRP3 being found to protect against dopaminergic neuronal loss in a similar toxin based model (Ou et al., 2020), further emphasising the NLRP3 inflammasome's role in neurodegeneration. Another example of NLRP3 modulation is via the use of kaempferol (Ka), a dietary flavonoid and phyto-oestrogen, which reduces NLRP3 inflammasome activation and protects against neurodegeneration, via upregulation of autophagy, in a murine A53T^tg/tg^ α-synuclein overexpressing model (Han et al., 2019). Ka has also been reported to reduce inflammation in a neuroinflammation model, using a murine microglial cell line (Park et al., 2011). However, this proposed mechanism is complicated by the fact that other work has implicated α-synuclein-mediated promotion of autophagy in activating the NLRP3 inflammasome in astrocytes extracted from mouse brain tissue; also use of an autophagy inhibitor, 3-methyladenine, led to decreased expression of NLRP3, caspase-1 and IL-1β (Wang et al., 2020)., Another *in vitro* model, using murine hippocampal HT22 cells, has demonstrated anti-inflammatory properties of the cyclosporine A derivative, N-methyl-4-isoleucine-cyclosporine (NIM811). NLRP3 inflammasome activation and cell death, via mitochondrial damage, was induced using rotenone and these effects were suppressed upon addition of NIM811 (Zhang et al., 2020). Hence, NLRP3 inhibitors warrant further pre-clinical investigation in PD.

## Huntington’s Disease

Huntington’s disease (HD) is an inherited autosomal-dominant disorder, characterised by progressive chorea, cognitive decline and psychiatric symptoms (Table 1). This neurodegenerative condition is caused by a CAG-trinucleotide repeat expansion in the huntingtin gene (*HTT*), that leads to production of defective huntingtin protein, which misfolds and accumulates within neurons, thus forming aggerates that affect normal cellular function (Soto, 2003; Williams and Paulson, 2008). Although our understanding of HD has grown in the last decade, existing treatments for HD are limited to treating only the symptoms of the disease, thus emphasising the urgent need to develop novel therapeutic approaches to treat this disease.

Neuroinflammation is a known phenomenon in HD and is likely to be involved in the pathophysiology of this condition (Soulet and Cicchetti, 2011; Crotti and Glass, 2015; Rocha et al., 2016; Palpagama et al., 2019). This neuroinflammation is believed to be driven by microglia and other cells within the brain, and unlike AD and PD, the presence of peripheral immune cells in the brain is not a typical finding in HD (Figure 1) (Sapp et al., 2001; Palpagama et al., 2019). Several oxidative stress and inflammatory markers are raised in the serum of patients with HD, including CRP, GM-CSF, TNF, IL-1β, IL-6 and IL-8, strongly suggesting an inflammatory phenotype in this neurological disorder (Björkqvist et al., 2008; Sánchez-López et al., 2012; Chang et al., 2015; Politis et al., 2015; Rodrigues et al., 2016). Further investigations have revealed localised brain inflammation in HD with high levels of TNF, IL-1β, IL-6 and IL-8 in several regions of the brain, including the striatum, cortex and cerebellum (Björkqvist et al., 2008; Silvestroni et al., 2009; Rodrigues et al., 2016). Interestingly, plasma levels of IL-18 were significantly reduced in patients with HD and also in the R6/2 HD mice model (Chang et al., 2015). Although IL-1β and IL-18 cytokines are typically released post activation of the NLRP3 inflammasome, the divergent levels of these two cytokines in HD suggest different roles. In fact, some studies indicate that different regulatory mechanisms control IL-1β and IL-18 secretion (Schmidt and Lenz, 2012; Zhu and Kannegant, 2017; Christgen et al., 2020), which is an important consideration in understanding their effects in several neurological disorders, including HD (Motta et al., 2007; Chang et al., 2015; Tzeng et al., 2018; De Biase et al., 2020).

The fact that IL-1β is raised in HD strongly suggests involvement of the NLRP3 inflammasome; one study has shown that *NLRP3* expression is significantly increased in peripheral blood mononuclear cells (PBMCs) from patients with HD when compared to healthy subjects (Glinsky, 2008). Siew et al. showed that galectin-3 is a critical mediator of the neuroinflammation observed in HD, which is driven by microglial cells via NF-κB and NLRP3 inflammasome-dependent pathways (Siew et al., 2019). Moreover, this study revealed that plasma levels of galectin-3 correlate with disease activity in patients with HD and also in HD mice models (Siew et al., 2019). Remarkably, galectin-3 KO in mice significantly increased the survival of mice with HD as well as reducing inflammation, huntingtin protein aggregation and motor dysfunction.

Activation of the unfolded protein response (UPR) in HD (Duennwald and Lindquist, 2008; Leitman et al., 2013; Kalathur et al., 2015) may be associated with the increased levels of IL-1β associated with induction of the NLRP3 inflammasome, as the NLRP3 inflammasome can be activated in both UPR-dependent and independent fashions (Menu et al., 2012; Oslowski et al., 2012). Activation of the NLRP3 inflammasome can be achieved by dimerisation and phosphorylation of inositol-requiring enzyme 1α (IRE1α) which, in turn, enables thioredoxin-interacting protein (TXNIP) to activate the NLRP3 inflammasome, resulting in caspase-1 cleavage and IL-1β secretion (Lerner et al., 2012; Abderrazak et al., 2015; Chen et al., 2018; Llanos-González et al., 2020).

There are still several knowledge gaps which need to be elucidated in the pathogenesis of HD, such as the origin of the neuroinflammation and whether inhibition of inflammation would effectively reduce the progression of this condition. It would be interesting to explore whether NLRP3 inhibition, or the use of other immunosuppressants, could reduce the pathophysiology of HD.

## Amyotrophic Lateral Sclerosis

Amyotrophic lateral sclerosis (ALS) is a fatal, adult-onset neurodegenerative disease characterised by a progressive degeneration of motor neurons within the brain stem, spinal cord and primary motor cortex (Table 1). Most ALS cases are sporadic (sALS), with familial ALS (fALS) contributing approximately 10% of cases (Ghasemi and Brown, 2018). Superoxide dismutase 1 (SOD1) mutations account for around 20% of fALS cases (Andersen, 2006) and represent the majority of animal models (Philips and Rothstein, 2015), the most common being a transgenic mouse expressing the human SOD1(G93A) mutant (Morrice et al., 2018). Neuroinflammation is increasingly associated with ALS pathogenesis (Liu and Wang, 2017), and, although there is evidence to implicate the NLRP3 inflammasome, its fundamental role remains unclear.

SOD1(G93A) mice display upregulated NLRP3, active caspase-1, IL-1β and IL-18 which correlate with dendritic swelling and neuronal loss in the brain (Figure 2) (Debye et al., 2018; Gugliandolo et al., 2018). Progression from pre-symptomatic to early-symptomatic ALS is associated with upregulated Nlrp3 and IL-1β gene expression, increased NLRP3 and ASC protein expression and mature IL-1β release in the SOD1(G93A) mouse spinal cord (Johann et al., 2015; Cunha et al., 2018), and NLRP3 protein expression, caspase-1 cleavage and mature IL-1β secretion in microglia is significantly increased in response to LPS (Bellezza et al., 2018). An increase in inflammatory markers is also seen in ALS patients; both sera and CSF samples exhibit significantly elevated IL-18 levels (Italiani et al., 2014), and caspase-1 levels in ALS patients’ sera are higher than in healthy controls (Iłzecka et al., 2001). However, evidence directly linking this inflammation to NLRP3 activity is limited. ALS patients’ monocytes show increased inflammatory gene expression, including *NLRP3* and *IL-18* (Zhao et al., 2017), and elevated *in-situ* expression of NLRP3, activated caspase-1 and IL-18 have been identified in post-mortem brain samples from sALS patients; however, it is unclear whether this is significant as they were compared to a single non-ALS counterpart (Kadhim et al., 2016). Increased levels of NLRP3, ASC and mature IL-1β have also been observed in human spinal cord tissue samples, although this did not reach significance levels in the case of NLRP3 (Johann et al., 2015).

One specific mechanism proposed to connect the NLRP3 inflammasome with ALS pathology is that misfolded proteins may act as inflammasome-stimulating DAMPs. Extracellular human SOD1(G93A) or SOD1(G85R), but not wild type SOD1, activates caspase-1 and causes mature IL-1β release when phagocytosed by mouse microglia and macrophages (Meissner et al., 2010; Zhao et al., 2010). TLR4 and CD14 pathways are required for this inflammatory cytokine release (Zhao et al., 2010), as is ASC (Meissner et al., 2010; Zhao et al., 2010), thus implicating an ASC-containing inflammasome. However, it is unclear whether NLRP3 is specifically involved. Meissner et al. found that caspase-1–mediated IL-1β release, in response to SOD1(G93A), occurs independently of LPS priming and NLRP3 (Meissner et al., 2010). By contrast, a recent study showed that NLRP3, expressed in microglia from SOD1(G93A) mice, is activated by aggregated and soluble SOD1(G93A) protein, leading to ASC speck formation, caspase-1 cleavage and mature IL-1β secretion. This SOD1(G93A)-mediated inflammation also involved ROS, ATP-mediated P2X7 receptor activation, with attenuation by the NLRP3-specific inhibitor MCC950, strongly suggesting that the NLRP3 inflammasome plays an essential role in the process (Deora et al., 2020).

Transactive response DNA-binding protein-43 (TDP-43) is also implicated in ALS pathology, as it forms a major component of intraneuronal aggregates in most ALS patients (Prasad et al., 2019), as well as triggering mtDNA release, acting as a trigger for the NLRP3 inflammasome (Yu et al., 2020). Extracellular TDP-43 causes activation of murine microglia and initiates a proinflammatory cascade featuring upregulation of NLRP3, active caspase-1 and mature IL-1β release. Co-culture of TDP-43-activated microglia with motor neurons causes motor neuron death, providing evidence that this protein may cause neurotoxicity via an NLRP3 inflammasome-related mechanism (Zhao et al., 2015). Conversely, the anti-inflammatory signaling hormone, 17β-estradiol, which has been linked to the lower incidence of ALS in women (de Jong et al., 2013), may have an inverse effect and improve motor performance by reducing NLRP3 inflammasome expression and function, with associated motor neuronal cell survival (Heitzer et al., 2017). TDP-43 has also been implicated in the onset and development of AD (Vanden Broeck et al., 2014; Budini et al., 2017), and various pathogenic mechanisms underlying AD, including the deposition of Aβ (LaClair et al., 2016; Davis et al., 2017), tau hyperphosphorylation (Davis et al., 2017; Gao et al., 2018), mitochondrial dysfunction (Izumikawa et al., 2017), and neuroinflammation (Herman et al., 2012); thus TDP-43 could also potentially trigger the NLRP3 inflammasome in AD, which warrants further investigation.

Evidence for the potential of the NLRP3 inflammasome as a therapeutic target in ALS varies between species. Knockout or inhibition of caspase-1, IL-1β or TLR4 delays symptomatic progression and mortality in SOD1(G93A) mice (Friedlander et al., 1997; Meissner et al., 2010; Lee et al., 2015), but does not affect disease onset, whereas the pan-caspase inhibitor, zVAD-fmk, delays both onset and mortality (Li et al., 2000). However, trials in humans with the recombinant IL-1 receptor antagonist, anakinra, have been less successful. In a case study of a patient suffering from severe idiopathic cold urticaria and ALS-linked neurological symptoms, anakinra caused remission of the CAPS-like symptoms but did not influence neurological symptoms (Bodar et al., 2009). Additionally, a pilot study assessing the safety of anakinra in 17 ALS patients found no overall difference in disease progression (Maier et al., 2015). This may be due to the dose used; 1–2 mg/kg was used in humans compared to 75–150 mg/kg in the mouse studies described above. As such, further studies assessing alternative IL-1 inhibitors with different therapeutic properties, as well as specific NLRP3 inhibitors, would help to assess the value of targeting this inflammasome in ALS (van der Meer and Simon, 2010).

## Prion Diseases

Prion diseases are a group of fatal neurodegenerative disorders of a genetic, sporadic or infectious nature, all of which are caused by misfolding of the PrP^C^ into a pathological isoform (PrP^Sc^). These diseases are characterised by spongiform degeneration, astrocytic gliosis, neuronal loss and the decay of cognitive function (Prusiner, 1998). Sporadic Creudzfeldt-Jakob disease (CJD) manifests between 55 and 75 years with rapidly progressing dementia, and several behavioral symptoms including delusions, hallucinations, depression, disorientation and memory loss (Table 1) (Chandra et al., 2016). Neuronal loss in CJD is mainly caused by an apoptotic event following the accumulation of misfolded prions (Giese et al., 1995; Gray et al., 1999a; Gray et al., 1999b). The number of apoptotic neurons has been shown to correlate with the number of activated microglia and, in turn, with the severity of neuropathological lesions (Van Everbroeck et al., 2002). In line with this, increased levels of inflammatory cytokines including IL-8, CCL2, TGFβ, TNF and IL-1β have been found in the CSF of sporadic CJD cases (Sharief et al., 1999; Van Everbroeck et al., 2002; Stoeck et al., 2006; Stoeck et al., 2014), with increased IL-1β levels correlating with the number of activated microglia at early stages of the disease (Van Everbroeck et al., 2002).

In 2012, two *in vitro* studies reported that aggregated/fibrillar PrP106-126 was involved in both priming and activation of the NLRP3 inflammasome (Figure 2) (Hafner-Bratkovic et al., 2012; Shi et al., 2012). In line with previous studies, aggregated PrP-peptide triggered NF-κB signaling, upregulating *IL-1β* expression as well as other components necessary for NLRP3 assembly (Hafner-Bratkovic et al., 2012). NLRP3 inflammasome activation and release of IL-1β in microglial cells was also reported to Increase extracellular K^+^ levels, and phagocytosis inhibition significantly attenuated PrP106-126-induced release of IL-1β, through downregulation of NLRP3 expression (Hafner-Bratkovic et al., 2012; Shi et al., 2012). PrP106-126 fibrils were also found to increase ROS production in treated microglia (Bacot et al., 2003; Yang et al., 2008). The ROS inhibitor, N-acetyl-l-cysteine (NAC), significantly reduced IL-1β production, and blocked NLRP3 and ASC upregulation after exposure to PrP106-126 in murine microglia (Shi et al., 2012). In a follow-up paper, these researchers found that the NLRP3 inflammasome complex negatively regulated TLR4-TRIF-mediated autophagy by activating caspase-1-induced TRIF cleavage in response to PrP106-126 stimulation (Lai et al., 2018). As chronic inflammation is a common feature of neurodegenerative diseases, the upregulation of autophagy by inhibiting caspase-1 activation, with reduced neuroinflammation and accelerated removal of misfolded protein, could be an attractive therapeutic strategy for prion-induced insults (Lai et al., 2018). This is consistent with the effect of Ka on reducing NLRP3 inflammasome activation via upregulation of autophagy in PD (Ahmed et al., 2020).

Despite the compelling *in vitro* data supporting the involvement of NLRP3 in the pathogenesis of prion diseases, a study by Nuvolone and colleagues showed that mice lacking NLRP3 (Nlrp3^−/−^) or the inflammasome adaptor protein ASC (Pycard^−/−^) succumbed to prion disease, with attack rates and incubation times similar to wild-type mice following inoculation with prions (strain RML) (Nuvolone et al., 2015). Levels of IL-1β at end-stage disease were not affected by the absence of NLRP3 or ASC proteins. This result does not directly contradict previous studies highlighting the importance of NLRP3/ASC inflammasome in the production of IL-1β (Agostini et al., 2004; Strowig et al., 2012; Lamkanfi and Dixit, 2014)*,* but does allude to the existence of other potential caspase-1 independent sources of IL-1β production, as seen in other disease phenotypes (Fantuzzi et al., 1997; Cheng et al., 2008). Nuvolone et al., 2015 argue that the *in vitro* generated PrP fibrils are not infectious and may trigger neurotoxicity by different pathways from those activated in prion infections; however, the discrepancies between these different groups may also be due to strain-dependent variations in prion infection, as shown by a number of different research groups, in both murine and human prion diseases (Baker et al., 1999; Tixador et al., 2010; Ayers et al., 2011).

Another factor which must also be considered is the influence of a primed inflammatory response in the course of prion diseases, whereby an underlying hyper-inflammatory state may be enough of a trigger to alter the clinical trajectory of these diseases. Interestingly, both Shi et al. (2012) and Hafner-Bratkovic et al. (2012) primed the cells with LPS to mimic chronic activation of microglia, as observed in prion diseases (Hafner-Bratkovic et al., 2012; Shi et al., 2012). Hafner et al. (2012) reported that while PrP-fibrils were able to activate NF-κB, and increase IL-1β mRNA, this activation might not be sufficient to produce prominent amounts of pro-IL-1β protein, but could produce enough to induce a primed state, which might be easily abrogated by bacterial infections or endogenous danger signals (Combrinck et al., 2002). However, it could be argued that infectious prions do not trigger inflammasome activation without an underlying inflammatory trigger. Therapies targeting the NLRP3 inflammasome shouldn’t be disregarded for prion diseases, although more *in vivo* studies, using a variety of infectious strains, are required to corroborate the *in vitro* data.

## Conclusion

Neuroinflammation, and its link to the progression of neurodegenerative disease, has been a key focus of research in the past decade, in the hope that eventual breakthroughs in this area will result in novel therapeutic approaches to treat these increasingly prevalent diseases. Despite the diverse range of mechanisms underlying these conditions, NLRP3 inflammasome activation and dysregulation are common features of several neurodegenerative diseases, both in the periphery and the CNS. Given the presence of an NLRP3 inflammasome signature in several neurodegenerative disorders, and that autoinflammatory diseases are primarily driven by NLRP3 inflammasome activation (de Torre-Minguela et al., 2017), it might be considered that these diseases are autoinflammatory-like in nature and therefore could be placed on the autoinflammatory immune spectrum (Peckham et al., 2017).

Although this review has focused on those inhibitors of the NLRP3 inflammasome that have already been studied in various neurodegenerative models, several other inhibitors are showing promising results in other disease models as well as in clinical trials. However, this topic is beyond the scope of this current review and the reader is referred to Caseley et al., 2020, for a more in-depth coverage (Caseley et al., 2020).

Several studies presented in this review have already shown that modulating NLRP3 inflammasome expression and activation inherent potential to delay the progression and impact of neuroinflammation in a number of neurodegenerative disease models, highlighting the importance of immune regulation. Given these promising results, NLRP3 modulators warrant further consideration for translation into clinical trials and may prove to have a common therapeutic benefit across a number of neurodegenerative disorders.
